# The AhR Ligand, TCDD, Regulates Androgen Receptor Activity Differently in Androgen-Sensitive *versus* Castration-Resistant Human Prostate Cancer Cells

**DOI:** 10.3390/ijerph120707506

**Published:** 2015-07-06

**Authors:** Maryam Ghotbaddini, Joann B. Powell

**Affiliations:** 1 Department of Biological Sciences, Clark Atlanta University, 223 James P. Brawley Drive, S.W. Atlanta, GA 30314, USA; E-Mail: maryam.ghotbaddini@students.cau.edu; 2 Center for Cancer Research and Therapeutic Development (CCRTD), Clark Atlanta University, 223 James P. Brawley Drive, S.W., Atlanta, GA 30314, USA

**Keywords:** TCDD/dioxin, AhR, androgen receptor, prostate cancer, castration-resistant

## Abstract

The reported biological effects of TCDD include induction of drug metabolizing enzymes, wasting syndrome and tumor promotion. TCDD elicits most of its effects through binding the aryl hydrocarbon receptor (AhR). TCDD induced degradation of AhR has been widely reported and requires ubiquitination of the protein. The rapid depletion of AhR following TCDD activation serves as a mechanism to modulate AhR mediated gene induction. In addition to inducing AhR degradation, TCDD has been reported to induce degradation of hormone receptors. The studies reported here, evaluate the effect of TCDD exposure on androgen receptor (AR) expression and activity in androgen-sensitive LNCaP and castration-resistant C4-2 prostate cancer cells. Our results show that TCDD exposure does not induce AhR or AR degradation in C4-2 cells. However, both AhR and AR are degraded in LNCaP cells following TCDD exposure. In addition, TCDD enhances AR phosphorylation and induces expression of AR responsive genes in LNCaP cells. Our data reveals that TCDD effect on AR expression and activity differs in androgen-sensitive and castration-resistant prostate cancer cell models.

## 1. Introduction

According to the Agency for Toxic Substances and Disease Registry (ATSDR) 2,3,7,8-tetrachlorodibenzo-p-dioxin (TCDD) is known to be a developmental toxicant in animals, resulting in kidney, skeletal and immune system defects in the offspring of animals exposed during pregnancy [1]. Because studies have shown an association between 2,3,7,8-TCDD and soft-tissue sarcomas, lymphomas, and stomach carcinomas in humans, the U.S. Environmental Protection Agency (EPA) has classified 2,3,7,8-TCDD as a probable human carcinogen (Group B2) [2,3]. Ambient measurements confirm that environmental TCDD contamination is widespread and it is the most toxic halogenated aromatic hydrocarbon [4]. There is significant concern about TCDD exposure from such diverse sources as municipal solid waste incinerators, pulp and paper mills, and contaminated fish and soil which could lead to serious health effects [3]. Dioxins consist of two benzene rings connected by two oxygen atoms and contain four to eight chlorines, for a total of up to 75 compounds or congeners [5]. Long-term TCDD exposure can lead to the development of tumors of the liver, thyroid, lung, prostate, skin, oral cavity, ovary and other sites in animal experiments [6,7]. TCDD is a multisite carcinogen and dioxins are extremely persistent and bioaccumulative. The half-life of TCDD in rodents is usually 2–4 weeks, but in humans it is estimated to be 7–11 years although with wide individual variation [5].

The aryl hydrocarbon receptor (AhR) mediates most toxic effects of dioxins [8]. The AhR is a basic helix-loop-helix ligand-activated transcription factor that mediates many of the responses to toxic environmental chemicals such as TCDD or dioxin-like PCBs. Ligand binding to AhR is shown to facilitate the release of HSP90 and its translocation to the nucleus [9]. To regulate gene expression, the AhR requires its binding partner, the aryl hydrocarbon receptor nuclear translocator (ARNT) and binds to specific gene regulatory sequences, called xenobiotic response elements (XRE’s) [10]. The high affinity ligand TCDD induces several AhR-mediated changes in gene expression, tissue/species-specific toxicities, and both tumorigenic and anticarcinogenic responses including inhibition of estrogen-dependent mammary and uterine tumor formation and growth [11]. After binding to XRE’s, the heterodimeric AhR/ARNT complex activates transcription of specific genes, including cytochrome P450s that are involved in xenobiotic compound metabolism. Induction of cytochrome P4501A1 (CYP1A1) gene expression by TCDD is considered to be an early and sensitive biochemical response and therefore it has been employed to measure exposure and sensitivity to this chemical [12,13].

Several mechanisms have been suggested to describe the AhR mediated interference of steroid hormone regulated responses, including altered hormone synthesis, enhanced ligand metabolism, down regulation of receptor levels and cross-talk between TCDD and estradiol or progesterone signal transduction pathways at the transcriptional level [9,14]. Specifically, androgen receptor (AR) is a member of the steroid hormone receptor family which is primarily responsible for mediating the physiological effects of androgens by binding to specific DNA sequences [15]. While it is possible that TCDD might interact directly with AR to produce androgenic influence, it might equally be able to interact with androgen-responsive cis–acting elements called androgen response elements (ARE) through ligand AhR/ARNT complexes. This type of cross-talk has been reported to occur with TCDD and estradiol in breast cells and may also play a dominant role in TCDD regulation of AR in prostate cancer [9,16].

The incidence of prostate cancer has risen during recent decades and it is now the most commonly diagnosed cancer among men in Europe and USA. It is the second most common cancer in men worldwide and a leading cause of mortality. Incidences continue to increase and vary considerably between populations [17].

Prostate cancer is slowly progressive with a long latency period. Such characteristics represent a good model of cancer in which to look for carcinogenic and chemopreventive agents [18]. Although the etiology of prostate cancer remains speculative, androgen receptor signaling is confirmed to play a pivotal role in development and progression. AhR activation by TCDD may increase prostate cancer risk, particularly in humans and animal models susceptible to prostate carcinogenesis [5]. AhR mediates the inhibitory effects of TCDD on prostate growth and also modulates normal prostate development [16,19]. Both *in vivo* and *in vitro* experiments suggest that TCDD, acting as an endocrine disruptor, may affect androgen receptor function and contribute to the development of prostate cancer [20,21]. However, our studies evaluate TCDD effects in androgen-sensitive and castration-resistant prostate cancer cell models.

The aim of these studies was to reveal differential regulation of androgen receptor expression and activity in an isogenic pair of prostate cancer cells that serve as models for progression to castration resistance. The androgen-sensitive LNCaP and castration-resistant C4-2 cell lines are used as a model system in these studies. This isogenic pair serves as an *in vitro* model of prostate cancer progression from hormone sensitive to hormone refractory. The castration-resistant C4-2 cells were derived from a chimeric tumor induced by inoculating a castrated mouse with the parental androgen sensitive LNCaP cells [22]. We previously reported that AhR is constitutively active in C4-2 cells and therefore TCDD may have differential effects in these two cell lines.

## 2. Materials and Methods

### 2.1. Reagents

Dimethyl sulfoxide (DMSO), R1881 and 3’4’-dimethoxyflavone (DMF) were purchased from Sigma-Aldrich (St. Louis, MO, USA). 2,3,7,8-tetrachlorodibenzo-p-dioxin (TCDD) was purchased from AccuStandard (New Haven, CT, USA). Antibodies to detect androgen receptor (sc-7305), phosphorylated androgen receptor (sc-52894), beta-actin (sc-81178), beta-tubulin (sc-55529) and topoisomerase (sc-271285) were purchased from Santa Cruz (Dallas, TX, USA). AhR antibody (ARP32243) was purchased from Aviva Systems Biology (San Diego, CA, USA).

### 2.2. Cell Culture

Adherent monolayer cultures of LNCaP and C4-2 human prostate cancer cell lines were maintained in RPMI 1640 medium supplemented with 10% FBS and 100 mmol/L each of penicillin and streptomycin. Cells were grown at 37 °C with 5% CO_2_ in humidified atmosphere, and media was replaced every third day. Cells were split (1:3), when they reached near confluence.

### 2.3. Protein Isolation and Western Blot Analysis

Protein samples were isolated using the NE-PER Extraction kit (Thermo Scientific, Waltham, MA, USA) for cellular fractions or commercially available cell lysis buffer (Cell Signaling, Boston, MA, USA) for total protein. Protein samples were resolved by SDS-PAGE and transferred to a PVDF membrane. Immunoblotting was carried out with 200 µg/mL mouse AhR monoclonal antibody at 1:500 dilution in 5% milk, 200 µg/mL mouse AR monoclonal antibody at 1:50 dilution in 5% milk, 100 µg/mL mouse pAR monoclonal antibody at 1:50 dilution in 5% milk, and 100 µg/mL rabbit Src or pSrc monoclonal antibody at 1:1,000 dilution in 5% BSA. Blots were washed three times (10 min each) with TBST. The blots were then incubated in 1:2,500 dilution of secondary antibody and washed three times (15 min each) with TBS. Bands were visualized with the enhanced chemiluminescence (ECL) kit as specified by the manufacturer. Multiple exposures of each set of samples were produced. The relative concentration of target protein was determined by computer analysis and normalized to an internal standard (β-actin).

### 2.4. Immunocytochemical Staining and Fluorescence Microscopy

Cells grown on glass cover slips in 6-well plates were washed in cold PBS and fixed by incubation in a 1:1 methanol: acetone solution at 4 °C for 30 min and then air dried. Cells were rinsed and hydrated with Tris-buffered saline containing 0.05% Tween 20 (TBST) and transferred to a clean 6-well plate. The cells were incubated at room temperature for 1 h in 5% milk solution in TBST to block nonspecific binding, followed by incubation at room temperature for 1 h with affinity-purified rabbit anti-AhR polyclonal antibody at 1 μg/mL at 1:1000 or 200 µg/mL mouse AR monoclonal antibody at 1:100 dilution in 4% milk solution in TBST. Cells were then washed three times (15 min each) with TBST. Cells were incubated with a 1:200 dilution of fluorescein isothiocyanate (FITC)-conjugated anti-rabbit antibodies (Jackson Immunoresearch laboratories, West Grove, PA, USA) or goat anti-mouse IgG-Rhodamine in 4% milk at room temperature for 1 h. The cells were then washed three times (15 min each) with TBST, three times (10 min each) with TBS and once with ddH20 (10 min). Cells were then mounted on slides using UltraCruz hard set mounting medium containing 4’6’-diamidino-2-phenylindole (DAPI).

### 2.5. RNA Extraction and Quantitative RT-PCR Analysis

Total RNA was isolated from cell monolayers grown in 100 mm tissue culture dishes using RNeasy Mini Kit (Qiagen, Valencia, CA, USA). Two µg of the total RNA was reverse-transcribed using the Superscript II kit (Invitrogen, Grand Island, NY, USA), according to the manufacturer’s recommendations. The cDNA served as a template in a 25 µL reaction mixture and was processed using the following protocol: an initial denaturation at 95 °C for 3 min, followed by 39 amplification cycles (95 °C for 10 s and 55–65 °C for 30 s), 95°C for 10 s, 65°C for 5 s and 95 °C for 50 s. The 25 µL qPCR reaction mixture was mixed with GoTaq qPCR Master Mix (Promega, Madison, WI, USA). Melt curve analyses were performed after each run to ensure a single product. Relative gene expression was determined using the ΔΔCq calculation method. The primer sequences used were: L-19: Forward (5′-3′) TCCCAGGTTCAAGCGATTCTCCTT and Reverse (5′-3′) TTGA GACCAGCCTGACCAACATGA; CYP1B1: Forward (5′-3′) TGCCTGTCACTATTCCTCATGCCA and Reverse (5′-3′) TCTGCTGGTCAGGTCCTTGTTGAT; KLK3 Forward (5′-3′) ACTTCAGTGT GTGGACCTCCATGT and Reverse (5′-3′) AGCACACAGCATGAACTTGGTCAC. Primers to amplify the 470-bp cDNA fragment encoding L-19 were used as an internal control.

### 2.6. Statistical Analysis

Statistical significance was evaluated using one-way ANOVA with multiple comparisons and Student’s *t*-test for two group comparison. The data are reported as mean ± SEM and a value of *p* < 0.05 was considered statistically significant.

## 3. Results

### 3.1. TCDDs Effect on AhR and AR Protein Expression in LNCaP and C4-2 Prostate Cancer Cells

Western blot analysis was used to evaluate AhR and AR protein expression in androgen-sensitive LNCaP and castration-resistant C4-2 prostate cancer cells in the presence of TCDD and R1881. LNCaP cells have higher AR protein levels that are diminished by TCDD exposure and enhanced by treatment with synthetic androgen R1881. TCDD treatment slightly reduced AhR protein levels in C4-2 cells while resulting in a 75% decreased in LNCaP cells.

**Figure 1:**
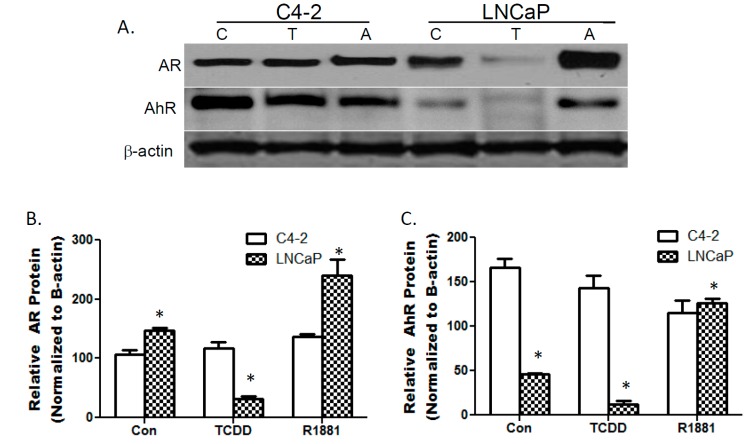
TCDD decreases AhR and AR protein expression in LNCaP cells. LNCaP and C4-2 cells were treated with DMSO (C), 10μM TCDD (T) or 10nM R1881 (A) for 24 h. Total protein transferred to a PVDF membrane was probed with AhR and AR antibodies (**A**). Bottom left panel is representative of AR protein expression (**B**) and bottom right panel of AhR protein expression (**C**). Image J was used to obtain densitometric measures from 3 independent membranes separately for AR and AhR. Each bar represents mean + SEM.

R1881 treatment also resulted in a modest decrease in AhR protein expression in C4-2 cells while significantly enhancing AhR expression in LNCaP cells. Overall, neither TCDD nor R1881 treatment altered AR expression in C4-2 cells (Figure 1).

### 3.2. TCDD Induces Nuclear Localization of AR in LNCaP but Not in C4-2 Prostate Cancer Cells

Immunohistochemical (IHC) staining confirmed previous reports that both AhR and AR are located in the cytoplasm and nucleus of C4-2 cells. TCDD induced AhR nuclear localization in LNCaP cells and further enhanced AhR nuclear localization in C4-2 cells. Cells were treated with 1 μM TCDD to preclude protein degradation that was previously induced with 10 μM treatment.

**Figure 2:**
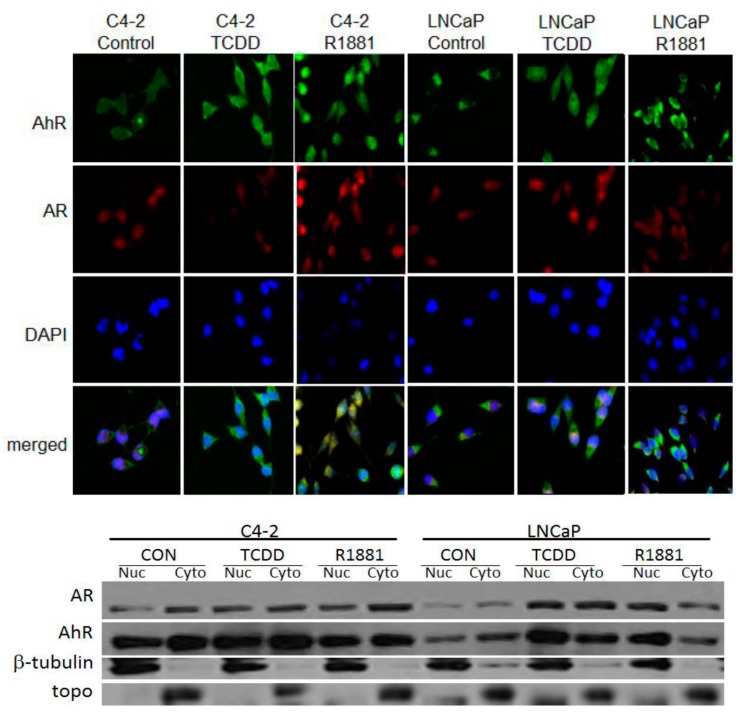
IHC staining of LNCaP and C4-2 prostate cancer cells. Cells were exposed to 1 μM TCDD or 10 nM R1881 for 24 h. AhR was visualized by staining with FITC-conjugated goat anti-rabbit antibody and AR with Rhodamine-conjugated rabbit anti-mouse antibody. The nuclei were counter-stained with DAPI fluorescence dye. Images from FITC, Rhodamine and DAPI-fluorescence channels were merged (**A**). Western blotting of nuclear (nuc) and cytoplasmic (cyto) fractions. Cells were exposed to 1 μM TCDD or 10 nM R1881 for 24 h and isolated fractions were transferred to a PVDF membrane which was probed with AhR and AR antibodies. β-tubulin was used as a cytoplasmic loading control and topoisomerase (topo) was used as a loading control for nuclear fractions (**B**).

Western blot analysis of cellular fractions revealed that TCDD significantly enhanced AR nuclear localization in LNCaP cells and also slightly enhanced AR nuclear localization in C4-2 cells. As expected, R1881 enhanced nuclear localization in both LNCaP and C4-2 cells. The synthetic androgen also induced a slight increase in AhR nuclear localization in LNCaP cells (Figure 2).

### 3.3. TCDD Enhances Expression of Androgen Responsive Gene KLK3 in LNCaP Cells

**Figure 3:**
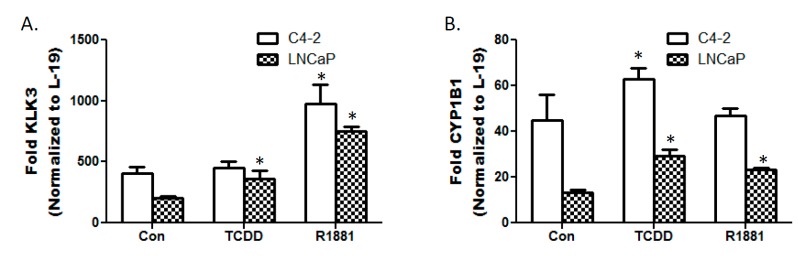
qRT-PCR analysis of KLK3 and CYP1B1 transcripts. Total RNAs were isolated and quantitative RT-PCR was performed to determine the mRNA expression of KLK3 and CYP1B1 in the prostate cancer cell lines. mRNA levels were normalized using L-19 which serves as an internal control. Each bar represents mean + SEM (n = 3). (*) denotes statistically significant differences between treatment groups and controls.

Androgen responsive gene KLK3 has increased expression in C4-2 cells compared to androgen sensitive LNCaP cells [23]. However, TCDD only enhances KLK3 expression in LNCaP cells. 10 M TCDD exposure for 24 h resulted in a 50% increase in KLK3 expression in LNCaP cells but did not affect expression in C4-2 cells. Androgen analog R1881 served as a positive control for induction of KLK3 expression in both LNCaP and C4-2 cells. Furthermore, R1881 enhanced expression of AhR responsive gene CYP1B1 in LNCaP cells. CYP1B1 was previously shown to be enhanced in C4-2 cells compared to LNCaP cells [24]. As expected AhR agonist TCDD enhanced CYP1B1 expression in both cell lines (Figure 3).

### 3.4. TCDD Induces AR Phosphorylation in LNCaP Cells

**Figure 4:**
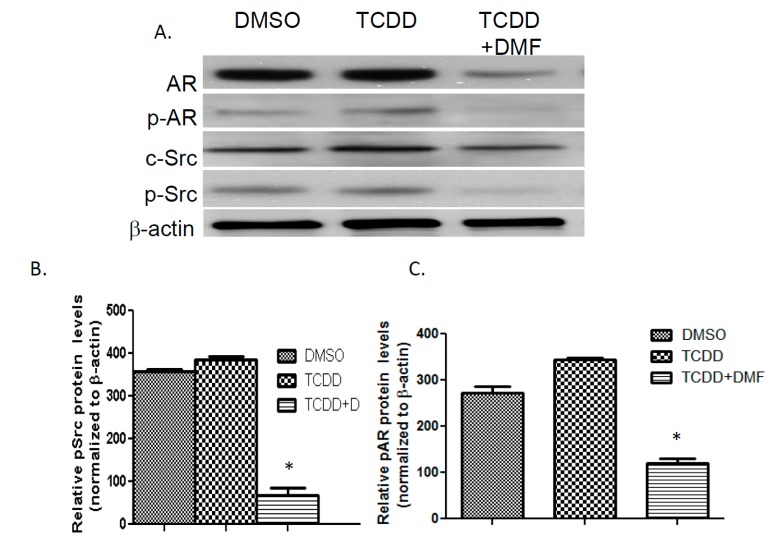
Western blot analysis of Src and AR phosphorylation. LNCaP cells were treated with DMSO, 1 μM TCDD or 10 μM DMF for 24 h. Total protein transferred to PVDF membrane was probed with AR, pAR, Src and pSrc antibodies (**A**). Bottom left panel is representative of pSrc protein expression (**B**) and bottom right panel of pAR protein expression (**C**). Image J was used to obtain densitometric measures from three independent membranes. Each bar represents mean + SEM.

To further examine TCDD’s effect on AR nuclear localization and transcriptional activity in LNCaP cells, we examined the effect of TCDD exposure on phosphorylation of AR and SRC kinase. Enhanced AR activity has been shown to correlate with Src kinase activity [25]. LNCaP cells were exposed to 1 μM TCDD for 12 h to prevent degradation of AR seen with higher concentrations at 24 h. TCDD exposure increased AR phosphorylation by more than 3-fold. Although the increase in phosphorylated Src kinase was not significant following TCDD exposure, AhR antagonist 3’,4’-dimethoxyflavone (DMF) diminished pSrc expression by more than 85%. In addition, DMF inhibited the ability of TCDD to induce phosphorylation of AR (Figure 4).

## 4. Discussion

The biological effects of TCDD include adaptive responses, such as the induction of drug metabolizing enzymes or toxic effects such as tumor promotion, wasting syndrome, and toxicity to the skin, immune, developmental, or endocrine systems [26,27,28,29]. Although the health effects of TCDD on the human population remain a matter of debate, TCDD is known to elicit most of its effects thorough the AhR. Additionally, numerous reports have shown that the AhR protein is rapidly depleted *in vitro* and *in vivo* following exposure to TCDD [30]. The TCDD-induced degradation of AhR requires ubiquitination of the AhR protein and serves as a mechanism for controlling the activity of the ligand activated receptor [31]. The down regulation of AhR has been observed in nine distinct cells culture lines derived from human and rodent tissues and has also been observed in rodent models following exposure to TCDD. The consequence of blocking AhR degradation in cell culture appears to be an increase in both the magnitude and duration of gene regulation by the AhR/ARNT complex. Thus, the physiological role of AhR degradation may be to modulate AhR-mediated gene regulation [32]. Our results show that TCDD exposure does not result in AhR degradation in the castration resistant C4-2 prostate cancer cells. This correlates to the sustained CYP1B1 expression seen prior to and following TCDD exposure.

In addition to inducing AhR degradation, there have also been reports of TCDD induced degradation of hormone receptors. Studies have shown the AhR ligand induced degradation of AR can be cell and compound specific. Treatment with AhR ligand benzo[α]pyrene (BaP) tremendously reduced constitutive AR expression as well as testosterone-induced AR protein levels in lung adenocarcinoma cells while TCDD did not reduce AR levels [33]. Dihydrotestosterone (DHT) stabilized AR protein in LNCaP cells that was subsequently decreased with TCDD cotreatment [34]. Transcription studies showed that activation of AhR blocks androgen-induced gene induction in LNCaP cells [35]. However our studies reveal that TCDD alone can induce androgen responsive genes in LNCaP cells. Furthermore, 24 h TCDD exposure decreased AR protein levels. Our data supports previous reports that AhR can act as an E3 ubiquitin ligase capable of degrading androgen receptor. A constructed constitutively active mutant AhR was shown to bind estrogen/androgen-responsive promoters with endogenous ERa/AR and through its E3 ubiquitin ligase activity promote proteasomal degradation of ERa/AR following transcription [36]. Our data shows that AhR does not retain the ability to degrade AR in C4-2 cells following TCDD exposure. Summarized the data suggest that TCDD induced degradation of AR may be dependent on endogenous AR activity.

In castration resistant C4-2 prostate cancer cells AR has sustained signaling [23]. Our data confirms the presence of AR in the nucleus of C4-2 cells as well as enhanced expression of AR responsive gene KLK3 without androgen stimulation. Previous studies have demonstrated antiandrogenic functions of TCDD. Transcriptional interference between TCDD and testosterone mediated signal transduction pathways has also been reported. However, these previous studies evaluated the ability of TCDD to ablate androgen stimulated AR activity [9]. Our studies evaluate the effect of TCDD on AR function without androgen stimulation. Our data reveals that TCDD effect on AR expression and activity differs in androgen sensitive LNCaP and castration-resistant C4-2 prostate cancer cells. Both AhR and AR proteins are resistant to TCDD induced degradation in C4-2 cells. Consequently, C4-2 cells also exhibit no induction of AR nuclear localization or KLK3 expression following TCDD exposure. TCDD significantly induces AR nuclear localization and KLK3 expression while subsequently diminishing AR protein levels in LNCaP cells. These results indicate that TCDD produces a greater androgenic response in androgen sensitive prostate cancer cells.

TCDD has been reported to induce rapid activation of c-Src kinase in several different cell lines [37]. c-Src protein kinase is associated specifically with the AhR complex in the cytosol and upon ligand binding to the Ah-receptor subunit, c-Src is activated and released from the complex [12]. Because Src mediated phosphorylation of AR resulted in nuclear localization, DNA binding and activation of AR in the absence of androgens [38,39], we evaluated the effect of TCDD on both c-Src and AR phosphorylation. Lower concentrations of 1 μM TCDD showed a slight enhancement of Src phosphorylation and a significant enhancement of AR phosphorylation. A lower concentration was necessary to prevent degradation of AR protein without the use of proteasome inhibitors. Previous studies have shown that the expression levels of AhR and AR were not affected by low dose exposure to TCDD for a 24 h time period [9].

Androgen sensitive prostate cancer cells have significantly more cytoplasmic AhR complex than the castration-resistant C4-2 prostate cancer cells [24]. The difference in cytoplasmic AhR complex and androgen receptor activity may account for the difference in response to TCDD exposure [40]. Dioxins have been shown to exert both androgenic and anti-androgenic effects on prostate development. TCDD exposure was shown to reduce ventral prostate weight through inhibition of AR activity. Yet, TCDD exposure also induced high levels of AR regulated transcripts at different stages of development [41]. More experiments are needed to define the relationship between AhR, AR and the differential effects of TCDD on prostate cancer at different stages. AhR may promote or inhibit androgen receptor activity depending on cellular content.

## 5. Conclusions

The most potent AhR ligand, TCDD, is classified as a known carcinogen and has been shown to increase overall mortality from cancer in numerous epidemiological studies. However, emerging evidence suggests that TCDD may inhibit androgen receptor signaling and prostate cancer progression. For example, TCDD inhibited testosterone-dependent transcriptional activity and testosterone-regulated prostate specific antigen (PSA) expression in a dose dependent manner. TCDD was also shown to block androgen dependent proliferation of prostate cancer cells. Simultaneous activation of AhR and AR with TCDD and an androgen derivative respectively, decreased AR protein levels. The data presented here compares the effect of TCDD exposure on AhR and AR activity in androgen sensitive LNCaP cells versus castration resistant C4-2 prostate cancer cells. Both AhR and AR proteins are resistant to degradation after TCDD exposure in C4-2 cells. In addition, TCDD exposure in C4-2 cells failed to stimulate androgen receptor activity and increase expression of KLK3. In contrast, TCDD induces androgen receptor nuclear localization and KLK3 expression in LNCaP cells. This induction is accompanied by diminished AhR and AR protein levels. This evidence suggests that the effect of dioxin exposure on prostate cancer cells differs in androgen sensitive prostate cancer cells compared to castrate resistant cells. TCDD appears to induce an androgenic response in androgen sensitive prostate cancer cells that is not seen in castration resistant cells. More studies are needed to determine the mechanism responsible for the diminished response to TCDD in castration resistant cells.
